# Different Benzendicarboxylate-Directed Structural Variations and Properties of Four New Porous Cd(II)-Pyridyl-Triazole Coordination Polymers

**DOI:** 10.3389/fchem.2020.616468

**Published:** 2020-12-17

**Authors:** Ying Zhao, Jin Jing, Ning Yan, Min-Le Han, Guo-Ping Yang, Lu-Fang Ma

**Affiliations:** ^1^College of Chemistry and Chemical Engineering, Luoyang Normal University, Luoyang, China; ^2^Key Laboratory of Synthetic and Natural Functional Molecule of Ministry of Education, Shaanxi Key Laboratory of Physico-Inorganic Chemistry, College of Chemistry & Materials Science, Northwest University, Xi'an, China

**Keywords:** coordination polymers, structural variations, topologies, gas adsorption, luminescent properties

## Abstract

Four new different porous crystalline Cd(II)-based coordination polymers (CPs), i. e., [Cd(mdpt)_2_]·2H_2_O (**1**), [Cd_2_(mdpt)_2_(*m*-bdc)(H_2_O)_2_] (**2**), [Cd(Hmdpt)(*p*-bdc)]·2H_2_O (**3**), and [Cd_3_(mdpt)_2_(bpdc)_2_]·2.5NMP (**4**), were obtained successfully by the assembly of Cd(II) ions and bitopic 3-(3-methyl-2-pyridyl)-5-(4-pyridyl)-1,2,4-triazole (Hmdpt) in the presence of various benzendicarboxylate ligands, i.e., 1,3/1,4-benzenedicarboxylic acid (*m*-H_2_bdc, *p*-H_2_bdc) and biphenyl-4,4′-bicarboxylate (H_2_bpdc). Herein, complex **1** is a porous 2-fold interpenetrated four-connected 3D ***NbO*** topological framework based on the mdpt^−^ ligand; **2** reveals a two-dimensional (2D) ***hcb*** network. Interestingly, **3** presents a three-dimensional (3D) rare interpenetrated double-insertion supramolecular net *via* 2D ···ABAB··· layers and can be viewed as an ***fsh*** topological net, while complex **4** displays a 3D ***sqc117*** framework. Then, the different gas sorption performances were carried out carefully for complexes **1** and **4**, the results of which showed **4** has preferable sorption than that of **1** and can be the potential CO_2_ storage and separation material. Furthermore, the stability and luminescence of four complexes were performed carefully in the solid state.

## Introduction

The coordination polymers (CPs) have gained considerable research interest by the self-assembly of various organic linkers (including different functional groups) with metal ions/clusters due to their special topological nets and broad applications (Wang et al., 2014, 2015; Smith et al., 2015; Wang and Wang, 2015; Dey et al., 2017; Hong et al., 2017; Islamoglu et al., 2017; Kariem et al., 2017; Lin et al., 2017; Li et al., 2018; Xing and Janiak, 2020; Zhang et al., 2020). Generally, the coordination preference of the nature of ligands and geometries of ions are the primary considerations in the preparation process of CPs. However, other factors, such as the choice of auxiliary ligands, pH, template effect, as well as reaction temperature, etc., are considered to be the great roles in the fabrications of the desired structures (Chen et al., 2015; Song et al., 2015; Waller et al., 2015; Blandez et al., 2016; Lannoeye et al., 2016; Manna et al., 2016; Rosa et al., 2016; Lu et al., 2017). Besides, the typical non-covalent supramolecular interlocks, including H-bonding, π···π stacking, etc., may also help to fine-tune the structural assemblies (Wheeler, 2013; Yadav and Gorbitz, 2013; Bhattacharya et al., 2014, 2016; DeFuria et al., 2016; Ju et al., 2016; Li et al., 2016, 2017; Song et al., 2016; Yao et al., 2016; Park et al., 2017). Thus, it is essential to explore these factors to further help study the relationship of structures and properties.

The current research findings have presented that N-containing heterocyclic ligands possess excellent binding abilities with different center metal ions to construct various CPs. Further, most of them may be acted as the neutral building units and also as the anionic units by releasing the acidic N-H groups of the ligands, showing more versatile coordination fashions (Lin et al., 2010, 2011; Chen et al., 2013; Li et al., 2019). Recently, there are some studies mainly focused on the N-heterocycles and their derivatives with five-membered rings (including the imidazole, triazole, or tetrazole groups, etc.) and multicarboxylate as mixed ligands to build different porous CPs (Pachfule and Banerjee, 2011; Chen et al., 2013; Liu et al., 2014; Qin et al., 2014; Wang et al., 2014; Du et al., 2015; Li et al., 2016, 2017; Yan et al., 2017), which often show exceptional gas sorption and luminescent performances. Similarly, the phenlycarboxylate tectons have also been proven to be the great candidates to yield functional CPs because of their abundant coordination modes with ions (Chen et al., 2013; Li et al., 2015; Jin et al., 2016; Wu et al., 2017).

As a combination of the above stated facts and our previous works, a rigid N-heterocyclic 3-(3-methyl-2-pyridyl)-5-(4-pyridyl)-1,2,4-triazole (Hmdpt) ligand was deliberately chosen with three kinds of phenyldicarboxylate linkers, i.e., 1,3/1,4-benzenedicarboxylate (*m*-H_2_bdc, *p*-H_2_bdc) and biphenyl-4,4′-dicarboxylate (H_2_bpdc) to prepare porous CPs in this study. The Hmdpt tecton has lots of coordination sites and also can be the excellent hydrogen bonding donor/acceptor in the assembly of new CPs. Herein, four new Cd(II)-CPs, [Cd(mdpt)_2_]·2H_2_O (**1**), [Cd_2_(mdpt)_2_(*m*-bdc)(H_2_O)_2_] (**2**), [Cd(Hmdpt)(*p*-bdc)]·2H_2_O (**3**), as well as [Cd_3_(mdpt)_2_(bpdc)_2_]·2.5NMP (**4**), were synthesized successfully. Complex **1** displays a 2-fold interlocked four-connected ***NbO*** framework; **2** reveals a two-dimensional (2D) ***hcb*** network. More interestingly, complex **3** presents a three-dimensional (3D) rare interlocked double-insertion ***fsh*** supramolecular net *via* 2D ···ABAB··· layers, while **4** is a 3D ***sqc117*** network. Their structural diversity indicates that the different secondary benzendicarboxylate linkers have great influence on the assembly process of new CPs. The stability and luminescence were investigated carefully for four complexes in the solid state. Especially, the different gas sorption performances have been carried out for **1** and **4** in detail, indicating **4** has preferable adsorption than that of **1** and can be the potential CO_2_ capture/separation material.

## Experimental Section

### Materials and General Methods

All the reagents/solvents were brought to use in the experiments with no further purification. The characterization methods of the four complexes are given in section Materials and General Methods. Because the synthesis processes of four CPs are very similar, thus the synthesis of CP **1** is only presented herein briefly, and others can be found in the supporting information.

Synthesis of [Cd(mdpt)_2_]·2H_2_O (**1**). A mixture of 3CdSO_4_·8H_2_O (0.05 mmol, 38.5 mg), Hmdpt (0.05 mmol, 11.8 mg), DMA (4 ml), and H_2_O (6 ml) was mixed in a 25-ml Teflon-lined stainless steel vessel, which was heated at 120°C for 72 h and then cooled to room temperature at the rate of 10°C/h to form colorless block crystals. Yield is 63% (based on Hmdpt). Elemental analysis of **1**, calculated (%): C 51.75, N 23.22, H 3.98; found: C 51.68, N 23.38, H 3.73. Fourier Transform Infrared spectra (FT-IR) (cm^−1^): 3,525 (m), 3,436 (m), 3,244 (m), 1,612 (m), 1,424 (m), 1,110 (s), 839 (w), 716 (w), 608 (s).

### X-Ray Crystal Structure Determinations

The instruments used for X-ray crystallographic measurements and the refinement methods and details by SHELXL-97 (Sheldrick, 1997) are displayed in section X-Ray Crystal Structure Determinations. The crystallographic data of four CPs are listed in Table 1. The Cambridge Crystallographic Data Centre (CCDC) numbers of the four CPs are 1543594–1543597, respectively.

**Table 1:** Crystal data and refinements of four complexes.

**Complexes**	**1**	**2**	**3**	**4**
Formula	C_26_H _24_ CdN_10_ O_2_	C_34_ H_2_8__ Cd_2_ N_10_ O_6_	C_21_ H_19_ CdN_5_ O_6_	C_66.5_ H_58.5_ Cd_3_ N_12.5_ O_10.5_
Mass	602.93	897.48	549.81	1,537.66
Crystal system	Trigonal	Monoclinic	Monoclinic	Monoclinic
Space group	*R*-3	*P*2_1_*/c*	*P*2_1_*/n*	*C*2*/c*
*a* [Å]	27.122 (4)	21.920 (2)	9.9364 (12)	30.332 (4)
*b* [Å]	27.122 (4)	7.1964 (8)	14.6653 (18)	11.0553 (13)
*c* [Å]	10.7626 (17)	30.860 (2)	15.4981 (19)	21.499 (3)
α [°]	90	90	90	90
β [°]	90	134.521(4)	103.739 (2)	114.604 (2)
γ[°]	120	90	90	90
*V* [Å^3^]	6856 (3)	3470.9 (5)	2193.8 (5)	6554.7 (14)
Z	9	1	4	4
*D*_calcd._[g·cm^−3^]	1.275	1.710	1.665	1.307
μ [mm^−1^]	0.747	1.286	1.044	1.015
*F* [000]	2646	1768	1104	2552
GOOF	1.069	1.182	1.014	1.019
*R*_1_*^*a*^* [I>2σ(I)]	0.0292	0.0418	0.0235	0.0488
*wR*_2_*^*b*^* (all data)	0.0699	0.1603	0.0840	0.1661

a R_1_ = ∑||F_o_|-|F_c_||/∑|F_o_|,

b *wR_2_ = [∑w(${\text{F}}_{\text{o}}^{2}$- ${\text{F}}_{\text{c}}^{2}$)^2^/ ∑w(${\text{F}}_{\text{o}}^{2}$)^2^]^1/2^*.

## Results and Discussion

### Structure Description of [Cd(mdpt)_2_]·2H_2_O (1)

**Figure 1:**
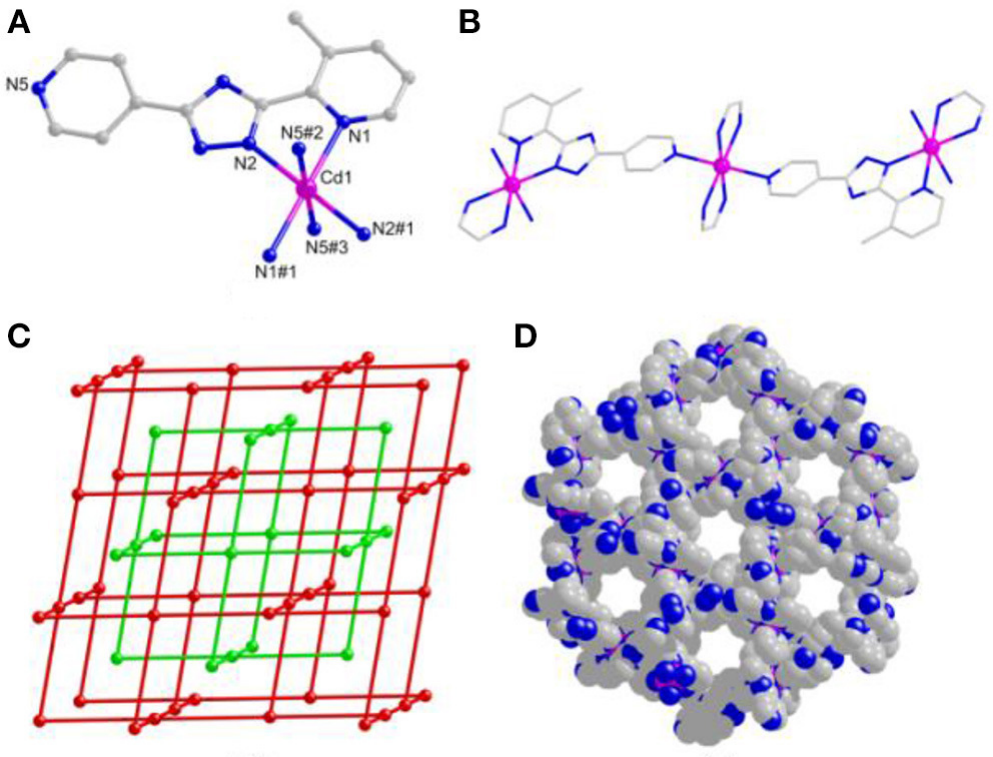
**(A)** The Cd(II) coordination geometry of **1**. **(B)** One-dimensional (1D) chain-like motif of **1**. **(C)** Three-dimensional (3D) space-filling view of **1**. **(D)** Topological representation of **1**.

Based on the X-ray single-crystal diffraction of complex **1**, it crystallizes in the *R*-3 trigonal system. Each Cd(II) ion is coordinated octahedrally with 2-pyridyl/triazolate N atoms from two mdpt^−^ linkers as the *trans* fashion as well as 4-pyridyl N atoms from the adjacent units (Figure 1A), and the similar complex of which has been reported before (Lin et al., 2010). The Cd–N lengths are the 2.215–2.408 Å, and the angles *via* Cd(II) center are 71.27° (8)−180.0°.

The mdpt^−^ linker holds two coordination fashions, i.e., bridged/chelated bidentate fashions ($\eta^{1}\mu_{1}\chi^{1}$ and $\eta^{2}\mu_{1}\chi^{2}$) in **1**, to coordinate with Cd(II) ions (Figure 1B and Supplementary Figure 4) to form a porous 3D framework (Figure 1C), and the void volume ratio of which is about 23.2% after exclusion of the guest water molecules by the calculation of PLATON program. Topologically, each ligand links two Cd(II) centers and each Cd(II) center joins four different bridged linkers, respectively. Thus, complex **1** displays a 2-fold four-connected interpenetrating ***NbO*** topological type with the (6^4^.8^2^) point symbol (Figure 1D).

### Structure Description of [Cd_2_(mdpt)_2_(*m*-bdc)(H_2_O)_2_] (2)

**Figure 2:**
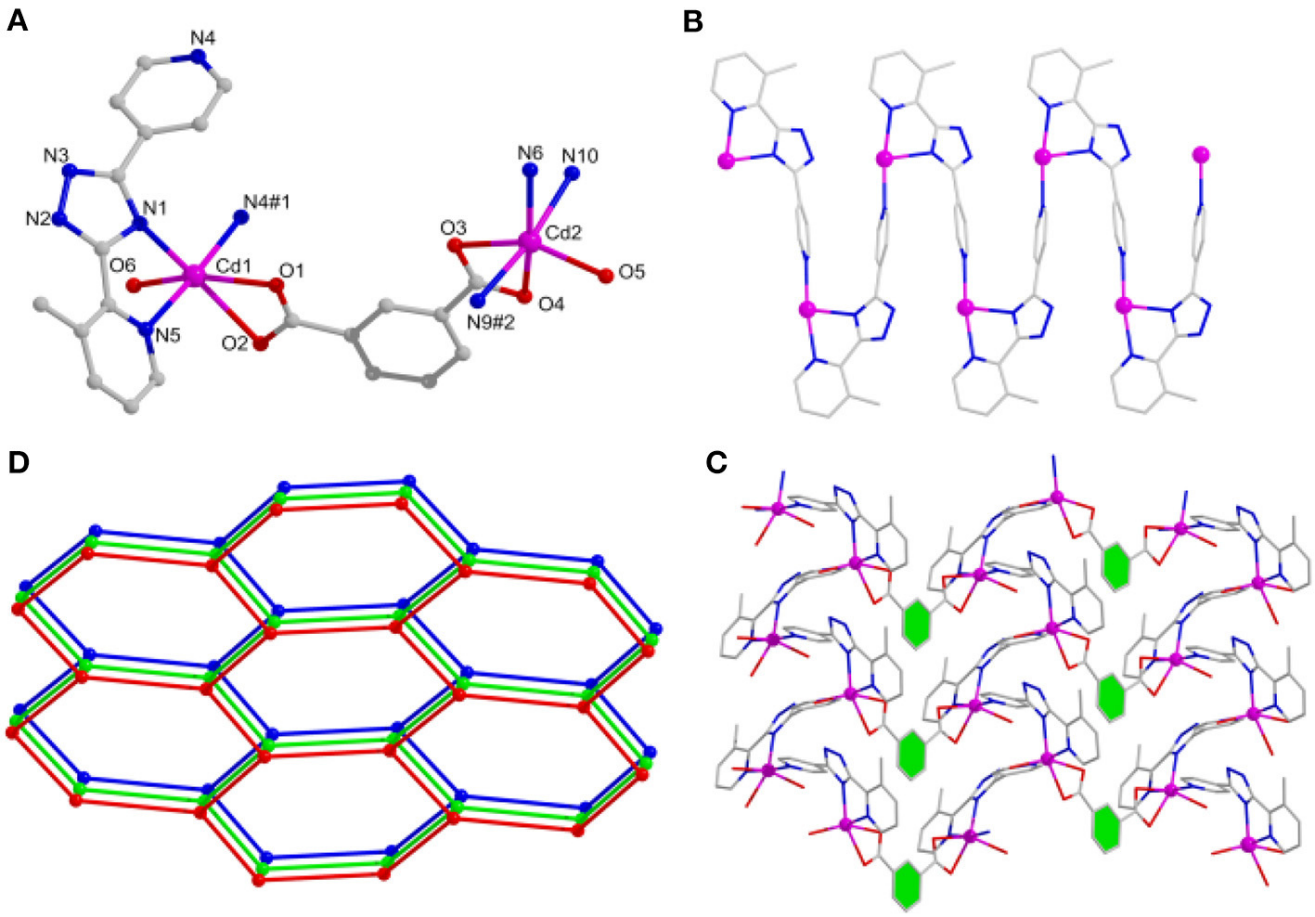
**(A)** The Cd(II) coordination environments of **2**. **(B)** One-dimensional (1D) chain motif of **2** along *a* axis. **(C)** Two-dimensional (2D) layered structure of **2**. **(D)** Topological net of **2**.

When the different carboxylate ligands are introduced in the reaction systems, Cd(II) centers are more prone to coordinate with O atoms than N atoms *via* the soft–hard acid–base (SHAB) principle, thus making various dimensionalities and structures of complexes (Zhang S. et al., 2013; Zhang X. et al., 2013). Herein, complex **2** has the *P*2_1_/*c* monoclinic system. The asymmetric unit holds two independent Cd(II) centers, two mdpt^−^, one *m*-bdc^2−^ linker, as well as two coordinated waters. All the Cd(II) ions have the hexa-coordinated geometries and possess the similar modes to link different ligands. Each Cd(II) center makes coordination with two O atoms of one *m*-bdc^2−^, one O atom of water molecule, and three N atoms of two different mdpt^−^ tectons (Figure 2A). The Cd–O/Cd–N distances are 2.272–2.434/2.267–2.371 Å, respectively; and the angles *via* Cd(II) ion are 55.49°-175.63° (Supplementary Table 1).

**Figure 3:**
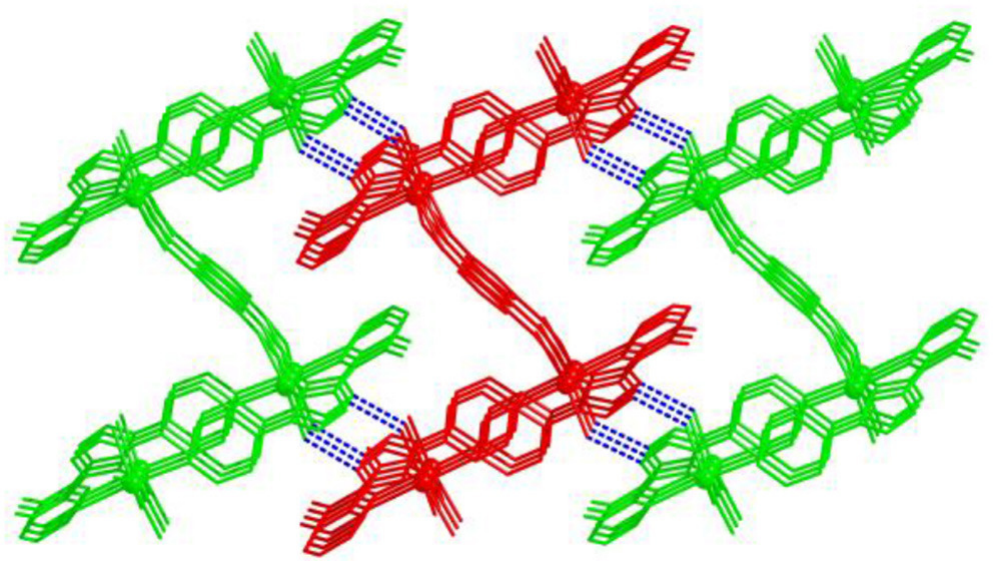
The intermolecular H-bonding interlocks between the two-dimensional (2D) layers, yielding a three-dimensional (3D) supramolecular motif along *c* axis.

In **2**, Cd(II) ions are connected through mdpt^−^ ligand to give rise to a 1D wave-like motif (Figure 2B). These adjacent 1D chains are further extended by *m*-bdc^2−^ tectons to yield a waved 2D layered structure along the *a* axis (Figure 2C). Topologically, each mdpt^−^ and *m*-bdc^2−^ linker two Cd(II) centers, which can be regarded as three-connected nodes, thus, the 2D structure can be viewed as a three-connected (6^3^) ***hcb*** net (Figure 2D). In addition, there exist the different hydrogen H-bonding interlocks (N_2_···O_5_, N_7_···O_6_) in the adjacent parallel 2D networks, which help to combine these 2D motifs as a final 3D supramolecular structure (Figure 3).

### Structure Description of [Cd(Hmdpt)(*p*-bdc)]·2H_2_O (3)

**Figure 4:**
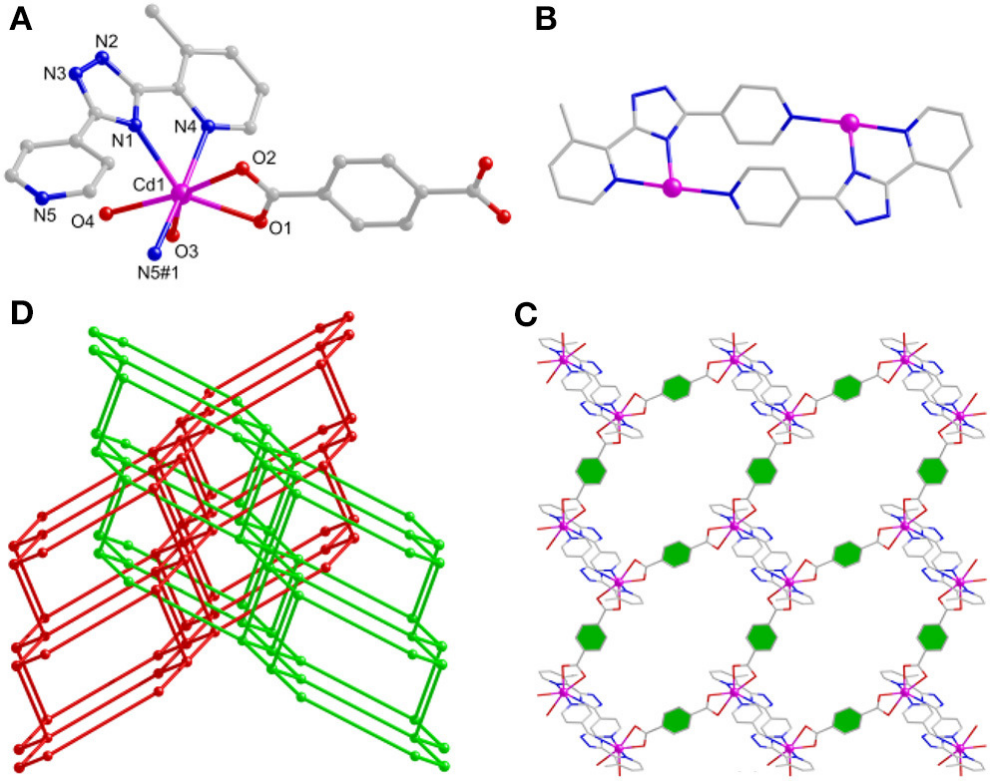
**(A)** The Cd(II) coordination geometry of **3**. **(B)** The binuclear unit of **3**. **(C)** The two-dimensional (2D) layered motif of **3** *via* the *a* axis. **(D)** The 2-fold interlocked topology of **3**.

Compared with the *m*-H_2_bdc ligand, the distance of the carboxylate groups of *p*-H_2_bdc is much farther to each other, which thus results in the various mode with Cd(II) centers and structure of **3**. Compound **3** has the *P*2_1_/*n* monoclinic system. The building block consists of a Cd(II) center, an Hmdpt, a *p*-bdc^2−^ tecton, and two guest waters. Each Cd(II) center adopts a seven-coordinated environment in which four O atoms are from the same *p*-bpc^2−^ and three N atoms are derived from two Hmdpt linkers (Figure 4A). The Cd–N/Cd–O lengths are 2.363~2.374/2.329~2.5175Å, and the angles *via* Cd(II) ion are 53.56–162.80° (Supplementary Table 1).

In **3**, the N atoms of Hmdpt ligand adopt 2-pyridyl and triazolate 1-nitrogens chelating and 4-pyridyl bridging modes with two Cd(II) ions, forming a secondary building unit [Cd_2_(mdpt)_2_] (Figure 4B), which produced an infinite 1D chain motif through *p*-bdc^2−^ ligand (Supplementary Figure 1). The neighboring 1D chains were successfully bridged by *p*-bdc^2−^ linkers to produce a new 2D layer via the *a* axis (Figure 4C). Like that of complex **2**, although **3** is also a 2D layered motif, however, the 2D layers are characterized by the accumulation of ···ABAB··· fashion. Further, the hydrogen bonds between the AA layers and water molecule O_6_ form a 3D supramolecular network and then interpenetrate with the 3D net held by the BB layers (Supplementary Figure 2). When the topological method is employed for analyzing the structure, complex **3** can be simplified as (4,6)-connected ***fsh*** framework, and the point symbol of which is (4^3^.6^3^)_2_(4^6^.6^6^.8^3^) (Figure 4D).

### Structure Description of [Cd_3_(mdpt)_2_(bpdc)_2_]·2.5NMP (4)

Due to the distance of two carboxylate groups of H_2_bpdc longer than those of **3**, thereby, a 3D dense framework of **4** is formed. Complex **4** crystallizes in the monoclinic *C*2*/*c crystal system. There contain three kinds of Cd(II) centers, two mdpt^−^ tectons, as well as two bpdc^2−^ linkers to form the asymmetric unit. The Cd–O/Cd–N distances are 2.185–2.613/2.229–2.395 Å, respectively.

**Figure 5:**
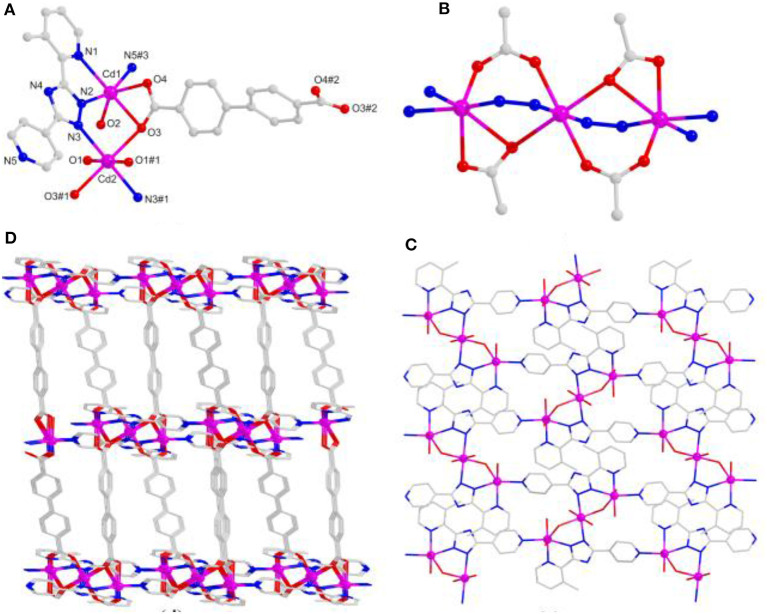
**(A)** The Cd(II) coordination geometries of **4**. **(B)** The [Cd_3_(μ_2_-COO)_4_N_4_] SBU of **4**. **(C)** The two-dimensional (2D) layered motif based on mdpt^−^ tectons of **4** via the *a* axis. **(D)** The three-dimensional (3D) porous structure of **4** via the *b* axis.

**Figure 6:**
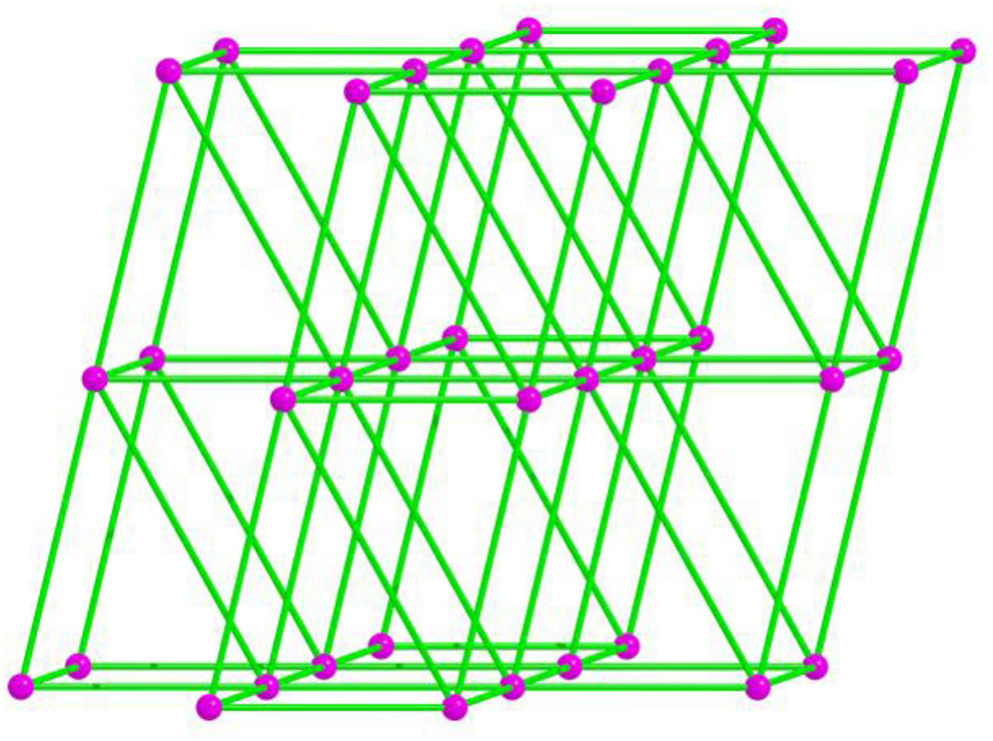
The topological framework of **4**.

Due to the twist of the two benzene rings, which makes the Cd1 and Cd2 centers adopt the same hexa-coordinated geometries but different coordination environments, where the Cd1 center takes coordination with three O atoms of bridging bpdc^2−^ tectons and three N atoms of mdpt^−^ linkers, the Cd2 center is bonded with four O atoms of bridging bpdc^2−^ tectons as well as two N atoms of mdpt^−^ linkers (Figure 5A). The deprotonated carboxylates exhibit $\eta^{2}\mu_{2}\chi^{3}$ and $\eta^{2}\mu_{2}\chi^{2}$ (Supplementary Figure 5) coordination modes and the N2, N3 of the mdpt^−^ ligands are coordinated with three Cd(II) ions to result in a new [Cd_3_(μ_2_-COO)_4_N_4_] secondary building unit (SBU) (Figure 5B). Such SBUs are further bridged by mdpt^−^ tectons to afford a 2D [Cd_3_(mdpt)_2_] layered motif along the *a* axis (Figure 5C). Moreover, these 2D adjacent motifs are further supported by introducing the flexible bpdc^2−^ linkers as the pillars to produce a 3D porous pillar-layered framework along the *b* axis (Figure 5D). As shown in Supplementary Figure 4, a pore space-filling structure is presented, and the void ratio is about 32.9% based on the PLATON program calculation. From a topological viewpoint, each Cd SBU may be simplified as the eight-connected nodes, and the overall net thus forms a (3^6^.4^12^.5^8^.6^2^) ***sqc117*** net (Figure 6).

From the above structural features of four CPs, it can be found that the different auxiliary tectons may give rise to the new structural variations and then further help to fine-tune the properties of CPs. In the same reaction systems, the minor differences of the ligands will make great changes in the assembly processes of CPs, the benefit of which should be explored carefully to control the fabrications of new CPs with desired structures and performances.

### Powder X-Ray Diffraction Measurement and Thermal Stabilities

The mass identities and phase purity of the four complexes were tested by comparisons of the experimental and the simulated powder X-ray diffraction (PXRD) patterns from the X-ray single-crystal data (Supplementary Figure 6), indicating the high-quality crystalline products.

The Thermogravimetric analysis (TGA) data were also tested carefully for studying the stability of the four complexes (Supplementary Figure 7). It is found that complex **1** firstly loses its guest waters in ~30–113°C [observed (obsd.) ~5.9%, calculated (calcd.) ~4.7%] and then collapse the linkers gradually. For complex **2**, a plateau is observed from the beginning to 30°C and then a ~3.3% loss in ~30–201°C, which matches well with the removal of two coordinated waters (calcd. ~4.0%); the second ~22% loss appears in ~315–366°C, which is attributed to a collapsed *m*-H_2_bdc ligand, and then the rest of the ligand starts to decompose gradually. Complex **3** loses guest waters in ~30–100°C (obsd. ~6.3%, calcd. ~6.6%), then follows a plateau of stability to ~306°C, and then an abrupt 29% loss in ~306–349°C, matching with the *p*-bdc loss (calcd. ~30%). Complex **4** incurs a ~15.4% loss in ~30–267°C for reduction in all NMP molecules (calcd. ~16.1%), then holds the stability to ~357°C.

### Gas Sorption of Complexes 1 and 4

**Figure 7:**
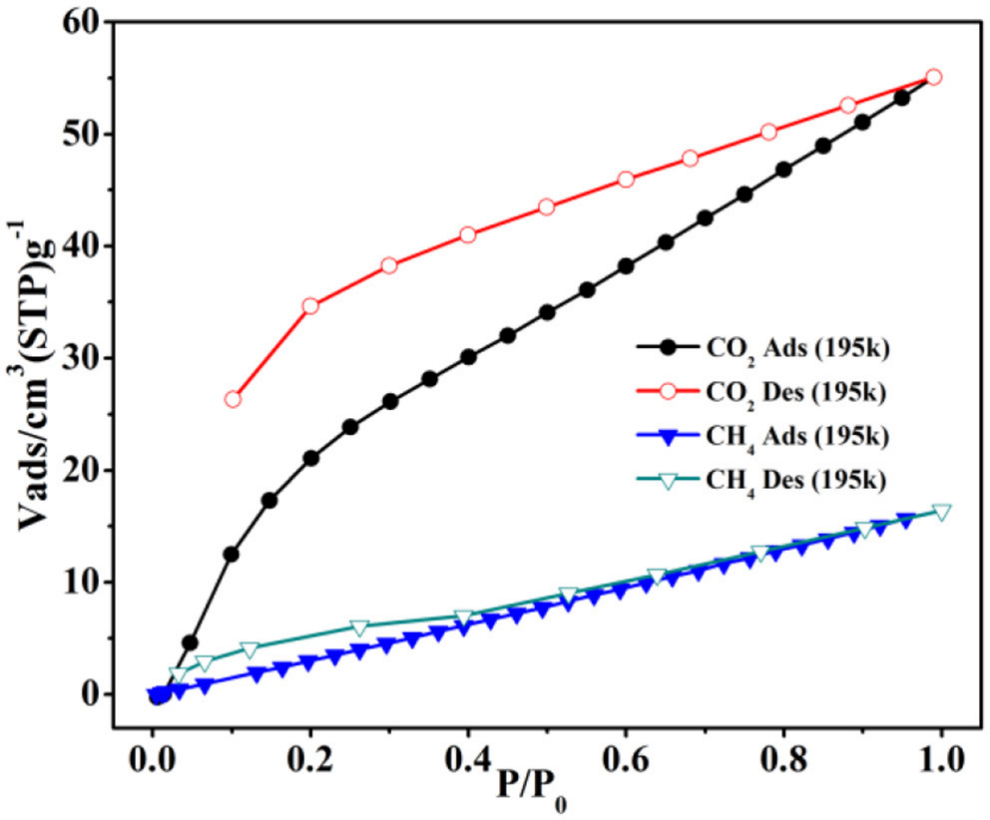
The CO_2_/CH_4_ sorption isotherms of **1a** at 195K.

The gas capture isotherms were tested carefully to prove the void properties of **1** for CO_2_ and CH_4_. The crystalline product of **1** was soaked in CH_2_Cl_2_ for 24 h and then dried under vacuum at 80°C to afford the desolvated **1a**. The gas captures of **1a** were performed carefully at 195 and 273K, respectively. At 1 atm, the uptake amount is 55.1 cm^3^ g^−1^ (1.08 wt.%) for CO_2_ (Figure 7), which is greatly higher than the CH_4_ uptake (16.4 cm^3^ g^−1^, 0.12 wt.%) at 195K. The gas sorption of CO_2_/CH_4_ was also performed at 273K (Supplementary Figure 9), and the CO_2_/CH_4_ uptake is 29.6/11.0 cm^3^ g^−1^ (0.58/0.08 wt.%) at 1 atm, respectively. Interestingly, the hysteresis effect is found for the CO_2_ sorption of **1a**, the reason of which might be the interactions of framework with CO_2_ to hinder the CO_2_ from the host framework in the sorption/desorption procedure (Biswas et al., 2013; Boldog et al., 2013; Liang et al., 2013; Han et al., 2017). Further, the PXRD patterns still closely matched well with the stimulated patterns of **1** (Supplementary Figure 6), indicating that the sample after heating retained the intact host framework.

Compared with complex **1**, **4** has preferable adsorption property, which may arise from introducing auxiliary H_2_bpdc ligand to give rise to more active sites and the flexibility of two benzene rings. Thus, the sorption isotherms were measured for N_2_/CO_2_/CH_4_ to verify the porosity of **4**. Herein, **4** is soaked in CH_2_Cl_2_ for 24 h and dried at 150°C under vacuum to yield the desolvated crystalline product (**4a**) successfully, which is proved by the combination of the TGA and FT-IR tests (Supplementary Figure 8). At 77K and 1 atm, the N_2_ uptake is 8.74 cm^3^ g^−1^ and the capture isotherm has an obvious hysteresis in the sorption/desorption process (Supplementary Figure 10).

**Figure 8:**
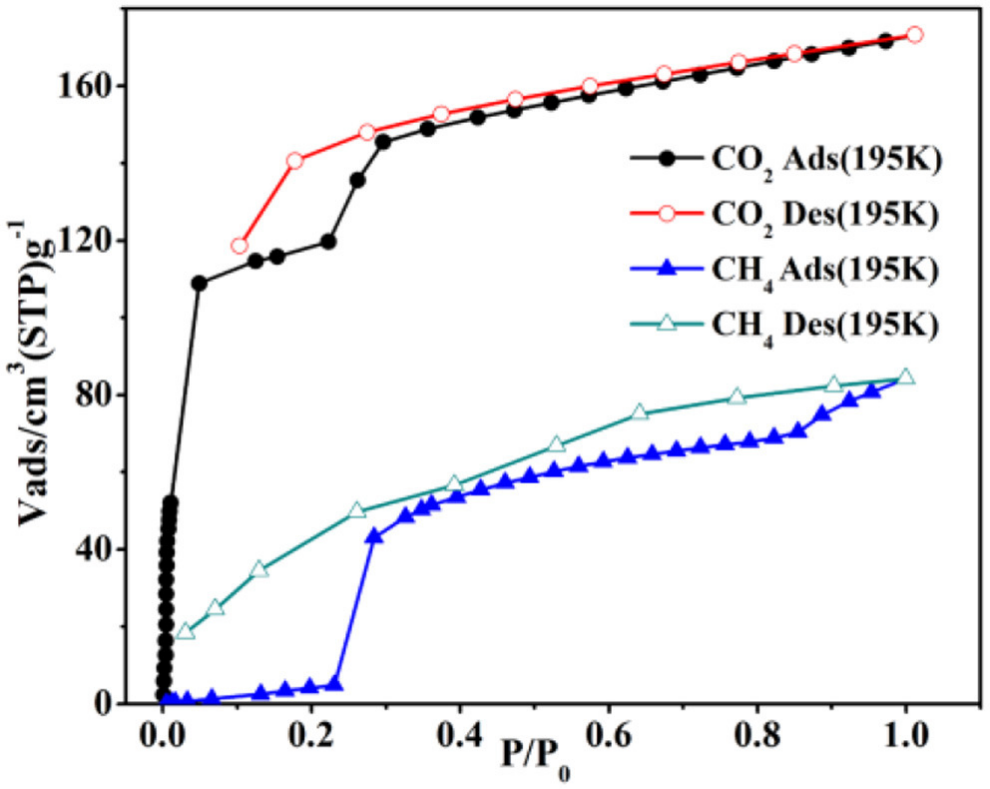
The CO_2_/CH_4_ sorption isotherms of **4a** at 195K.

At 195K and 1 atm, the CO_2_ sorption has a higher capture amount (34.04 wt.%, 173.28 cm^3^ g^−1^) to further prove the pore performances of **4a**. The Brunner-Emmet-Teller (BET) and Langmuir surface areas are 273.65/382.56 m^2^ g^−1^. Interestingly, the CO_2_ sorption exhibits a double abrupt increase at 0.04, 0.22 atm as a significant hysteretic desorption curve (Figure 8). In the initial step, the CO_2_ sorption amount is adsorbed as 109 cm^3^ g^−1^ (21.41 wt.%) for **4a**, and then the isotherm has a sudden increase in the second step (*P*/*P*_0_ = 0.2) and finally keeps the saturation. The desorption process does not retrace the capture process, which shows the different hysteresis, and the amount is 119 cm^3^ g^−1^ (*P*/*P*_0_ = 0.1) at the end of desorption. The incomplete desorption suggests there exist the interactions of CO_2_ and pore surface. Generally, this phenomenon in rigid metal-organic framework (MOFs) may be ascribed to the sorbate/sorbent interlocks as the gases capture by surfaces of the structure, obtaining the various pores as it breathes (Bezuidenhout et al., 2015; Ichikawa et al., 2016; Esfandiari et al., 2017). When at relatively lower pressures, the two distorted benzene groups of bpdc^2−^ ligand make the pore open, thus, leading to a rather large hysteresis.

The sorption isotherm of CH_4_ shows a lower amount (6.02 wt.%, 84.19 cm^3^ g^−1^) at 195K and 1 atm (Figure 8). However, it has a more obvious abrupt step with two sudden increases at 0.22/0.85 atm, which is similar to the CO_2_ isotherm. The CO_2_ is captured as 3.08 wt.% (43.23 cm^3^ g^−1^) for **4a** in the initial step, and then the isotherm holds a sharp increase (*P*/*P*_0_ > 0.85) and finally keeps the saturation. And the desorption process also displays the several steps at the corresponding points. As a result, the large hysteresis loops happen, leading that the CH_4_ is packed in the structure at the lower pressures and not released right now on decreasing the external pressure (Liu et al., 2013; Handke et al., 2014; Ju et al., 2015; Hiraide et al., 2017; Sun et al., 2017).

**Figure 9:**
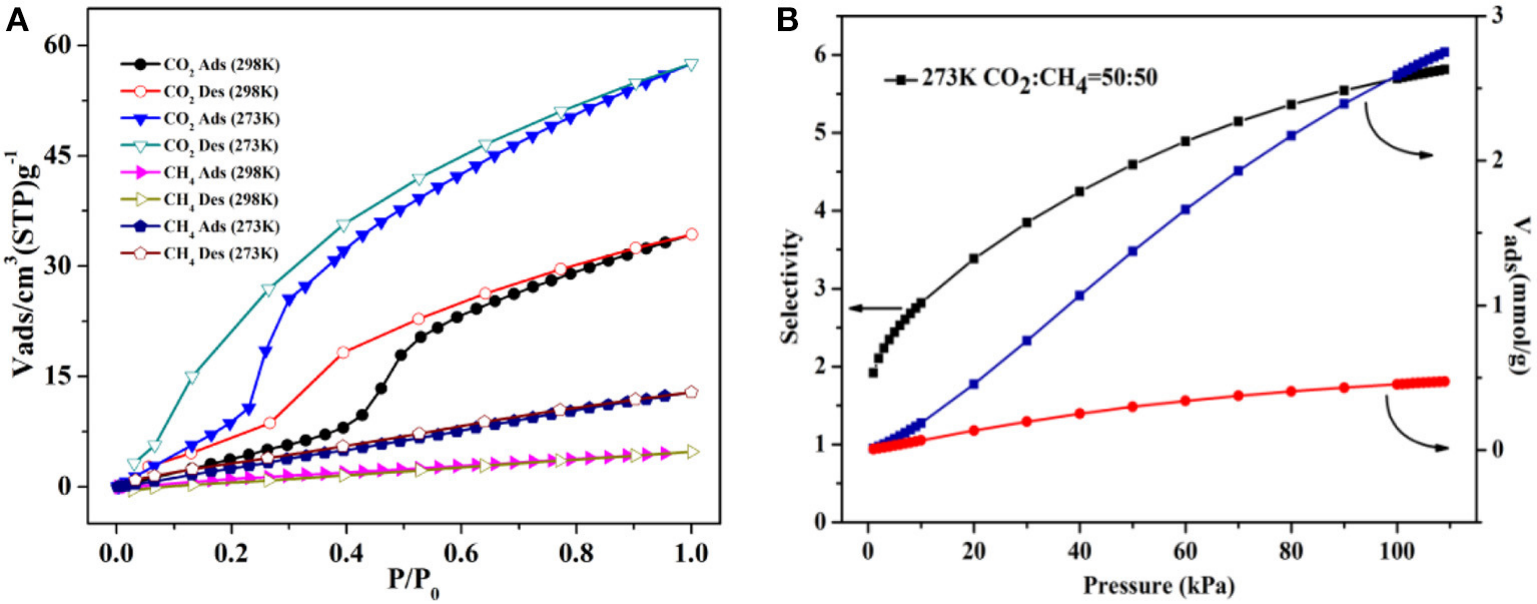
**(A)** The CO_2_/CH_4_ isotherms of **4a** at 273/298K. **(B)** The ideal adsorbed solution theory (IAST) selectivity for equimolar CO_2_/CH_4_ mixture at 273K.

The CO_2_/CH_4_ sorption properties of **4a** were also explored at 273/298K. The CO_2_ uptake amounts are 57.53 cm^3^ g^−1^ (11.3 wt.%) and 34.28 cm^3^ g^−1^ (6.7 wt.%) at 273/298 K, 1 atm (Figure 9A), and the CH_4_ amounts are 12.83 cm^3^ g^−1^ (0.9 wt.%) and 4.75 cm^3^ g^−1^ (0.3 wt.%) at 273/298K. These results indicate that **4a** has the selectivity for CO_2_ molecules, which is more evident at the higher temperature. Thus, the CO_2_/CH_4_ selectivity of **4a** is calculated by ideal adsorbed solution theory (IAST) at 273K (Chen et al., 2015) (Supplementary Figure 11). Here, the CO_2_/CH_4_ molar fraction is set as 50/50 to simulate the content of biogas mixture (Figure 9B). The results present that CO_2_ selectivity rapidly ascends with increasing load for both mixed contents over CH_4_, and the selectivity CO_2_/CH_4_ is calculated as 5.7 from the equimolar gas phase mixture at 1 atm.

**Figure 10:**
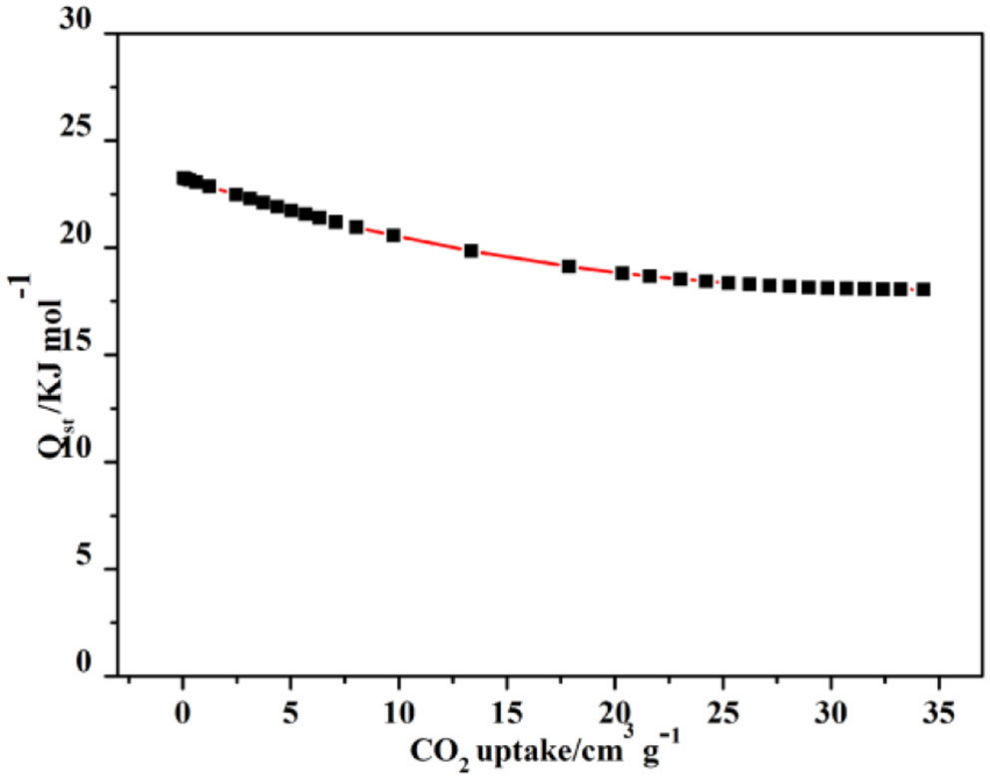
The CO_2_ Q_st_ of **4a** at 273K.

The capture enthalpy (Q_st_) was estimated to explore the CO_2_ affinity via the Virial equation of sorption isotherms at 273K (Supplementary Figure 12), revealing **4a** has a higher CO_2_ affinity at high loading amount. The Q_st_ has a slow decrease by the CO_2_ increasing capture; however, it is 23.2 kJ mol^−1^ at the final (Figure 10). This value is comparable to those of the hydrated HKUST-1, MAF-2, JUC-132, JLU-Liu, and NOTT-140 (30, 27, 30, 30, and 25 kJ/mol), but higher than those of most “benchmark CPs,” such as CuBTTri, MOF-5, and UMCM-1 (21, 17, 12 kJ/mol) (Wang et al., 2015) Thereby, complex **4** may be explored as a great gas adsorbent in many fields.

### Luminescent Properties

**Figure 11:**
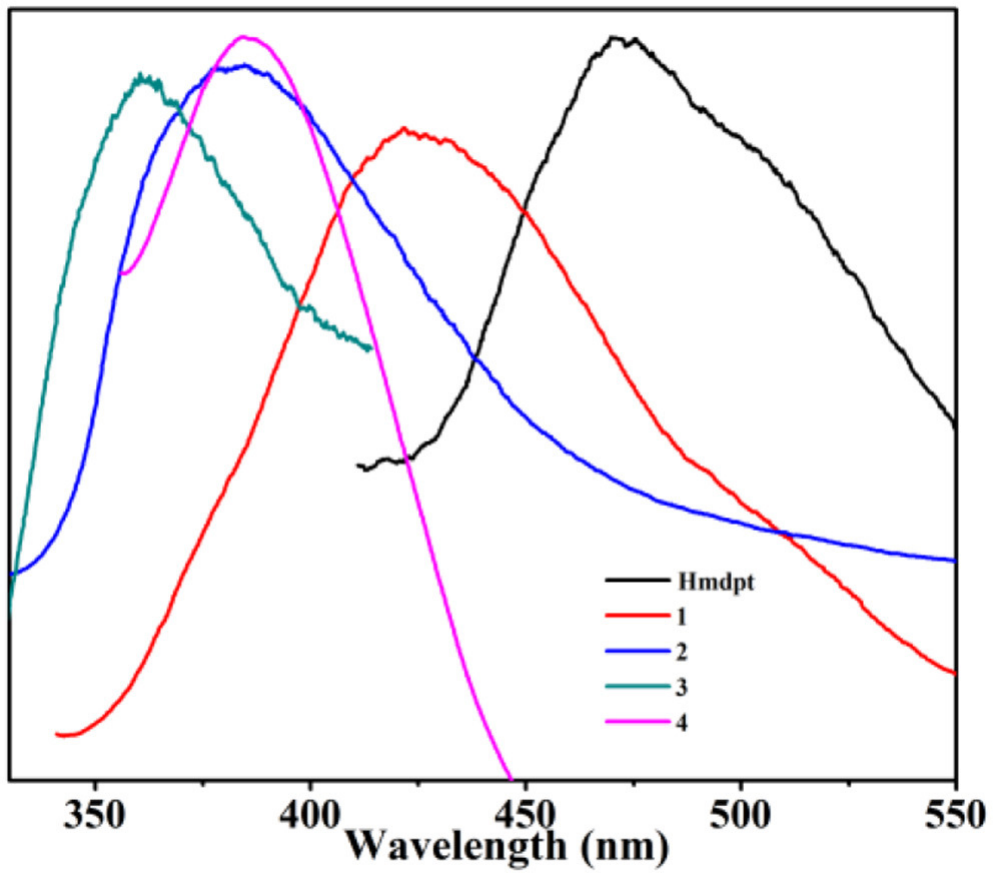
The solid-state luminescent emission spectra of the four coordination polymers (CPs) and Hmdpt linker.

Due to the great luminescent performances of the Hmdpt linker and d^10^ complexes (Chou et al., 2014; Kumar et al., 2014; Hu et al., 2015; Ye et al., 2015; Wang et al., 2016; Hong et al., 2017; Lim et al., 2017), the solid-state photoluminescence properties have been studied for the four complexes and Hmdpt as well as the various auxiliary benzendicarboxylates at room temperature (Figure 11 and Supplementary Figure 13), the emissions of which are given in Supplementary Table 2. The free Hmdpt shows the band at 472 nm when excited at 307 nm, attributing to the π^*^ → π transition of the intra-ligand (Lin et al., 2017). The four complexes have their specific emissions, the bands of which were presented at 425 nm (λ_ex_ = 321 nm) for **1**, 380 nm (λ_ex_ = 305 nm) for **2**, 360 nm (λ_ex_ = 289 nm) for **3**, as well as 386 nm (λ_ex_ = 336 nm) for **4**, respectively. These emission bands are greatly similar to the free Hmdpt emission for the π–π^*^ or *n*–π^*^ intra-ligand transition (Sun et al., 2013; Wenger, 2013; Zou et al., 2013; Chen et al., 2017). In contrast to the Hmdpt, the emissions of four CPs have the similar blue shifts, which are considered to be the energy transfer from the Hmdpt ligand to the Cd(II) centers for the ligand-to-metal charge transfer (LMCT) (Cao et al., 2017).

## Conclusion

In summary, four new CPs with rigid Hmdpt and different benzendicarboxylate linkers have been successfully obtained. The different auxiliary carboxylates resulted in a series of new various structural CPs. Complex **1** has a 2-fold interlocked ***NbO*** net; **2** presents the 2D three-connected ***hcb*** net; **3** shows the double-insertion 3D ***fsh*** supramolecular network; and **4** displays a 3D eight-connected ***sqc117*** net. The CH_4_/CO_2_ sorption behaviors of **1** and **4** have been carefully carried out at different temperatures. Remarkably, complex **4** has preferable sorption and high CO_2_ selectivity, making it as a useful gas storage/separation functional material.

## Data Availability Statement

The datasets presented in this study can be found in online repositories. The names of the repository/repositories and accession number(s) can be found in the article/Supplementary Material.

## Author Contributions

All authors listed have made a substantial, direct and intellectual contribution to the work, and approved it for publication.

## Conflict of Interest

The authors declare that the research was conducted in the absence of any commercial or financial relationships that could be construed as a potential conflict of interest.
