# Supplementary material for: Different Benzendicarboxylate-Directed Structural Variations and Properties of Four New Porous Cd(II)-Pyridyl-Triazole Coordination Polymers
Source: Front Chem. 2020 Dec 17;8:616468. doi: 10.3389/fchem.2020.616468 (PMC7773847; doi:10.3389/fchem.2020.616468)
Supplement: Supplementary file 1 [file Data_Sheet_1.docx]

**Supporting Information**

**Different Benzendicarboxylate-directed Structural Variations and Properties of Four New Porous Cd(II)-pyridyl-triazole Coordination Polymers**

***Ying Zhao ^1†^, Jin Jing ^2†^, Ning Yan ^2^, Min-Le Han ^1^, Guo-Ping Yang^2*^ and Lu-Fang Ma ^1*^***

*^1^ College of Chemistry and Chemical Engineering, Luoyang Normal University, Luoyang 471934, P. R. China*

*^2^ Key Laboratory of Synthetic and Natural Functional Molecule of Ministry of Education, Shaanxi Key Laboratory of Physico-Inorganic Chemistry, College of Chemistry & Materials Science, Northwest University, Xi'an 710127, P. R. China.*

*E-mail: [mazhuxp@126.com](mailto:mazhuxp@126.com); [ygp@nwu.edu.cn](mailto:ygp@nwu.edu.cn).

†These authors have contributed equally to this work

**Section S1 Materials and general methods**

All chemical reagents were purchased and used without further purification. Elemental analysis for C, H and N were carried out by Perkin-Elmer 2400C Elemental Analyzer. The infrared (IR) spectra were acquired with KBr dics in the range of 4000 to 400 cm^-1^ via Bruker Equinox-55 FT-IR spectrometer. The thermogravimetric analyses (TGA) were collected on Netzsch STA 449C microanalyzer thermal analyzer under N_2_ stream at a heating rate of 5 °C min^-1^. Powder X-ray diffraction (PXRD) data were measured by Bruker D8 Advance X-ray power diffractomer. All the gas sorption isotherms were measured by ASAP 2020 M adsorption equipment with an automatic volumetric sorption apparatus. Luminescence spectra measurements were recorded on Hitachi F-4500 luminescence spectrometer.

**Synthesis of [Cd_2_(mdpt)_2_(m-bdc)(H_2_O)_2_] (2)**

A mixture of 3CdSO_4_·8H_2_O (0.05 mmol, 38.5 mg), Hmdpt (0.05 mmol, 11.8 mg), m-H2bdc (0.05 mmol, 8.3 mg), H_2_O (4 mL), and DMF (6 mL) were mixed in 15 mL Teflon-lined stainless steel vessel, which were heated at 120 °C for 72 h and then cooled to the room temperature at the rate of 10 °C/h to form colorless block crystals. Yield 55% (based on Hmdpt). Elemental analysis of **2**, calculated (%): C 45.46, N 15.60, H 3.12; found: C 45.48, N 15.53, H 3.15. FT-IR (cm^-1^): 3421 (m), 2927 (w), 2471 (w), 1604 (m), 1374 (s), 1110 (w), 1002 (w), 860 (m), 716 (m), 575 (w).

**Synthesis of [Cd(Hmdpt)(p-bdc)]·2H_2_O (3)**

A mixture of 3CdSO_4_·8H_2_O (0.05 mmol, 38.5 mg), Hmdpt (0.05 mmol, 11.8 mg), *p*-H_2_bdc (0.05 mmol, 8.3 mg), H_2_O (4 mL), and NMP (6 mL) were mixed in 15 mL Teflon-lined stainless steel vessel, which were heated at 120 °C for 72 h and then cooled to the room temperature at the rate of 10 °C/h to form colorless block crystals. Yiel 63% (based on Hmdpt). Elemental analysis of **3**, calculated (%): C 45.83, N 12.73, H 3.46; found: C 45.87, N 12.63, H 3.51. FT-IR (cm^-1^): 3421 (m), 3229 (m), 3049 (m), 2927 (m), 1612 (m), 1514 (s), 1318 (s), 1110 (w), 1002 (m), 832 (s), 716 (s), 575 (w), 517 (m).

**Synthesis of [Cd_3_(mdpt)_2_(bpdc)_2_]·2.5NMP (4)**

A mixture of 3CdSO_4_·8H_2_O (0.05 mmol, 38.5 mg), Hmdpt (0.05 mmol, 11.8 mg), H_2_bpdc (0.05 mmol, 12.1 mg), H_2_O (3 mL), and NMP (3 mL) were mixed in 15mL Teflon-lined stainless steel vessel, which were heated at 150 °C for 72 h and then cooled to the room temperature at the rate of 10 °C/h to form colorless block crystals. Yield 65% (based on Hmdpt). Elemental analysis of **4**, calculated (%): C 51.90, N 11.38, H 3.80; found: C 50.96, N 11.40, H 3.67. FT-IR (cm^-1^): 3436 (w), 2927 (w), 1691 (m), 1590 (s), 1532 (m), 1381 (s), 1110 (m), 1002 (m), 846 (m), 774 (m), 724 (m), 673 (m), 546 (w).

**Section S2 X-ray Crystal Structure Determinations.**

Single-crystal X-ray diffraction data of complexes **1**–**4** were collected on the Bruker SMART APEX II CCD diffractometer equipped with a graphite-monochromated Mo-Κα radiation (λ = 0.71073 Å) at 296 K. All structures were solved by direct methods and refined by full-matrix least-squares fitting on *F*^2^ by SHELXL-97. The absorption data were corrected by utilizing *SADABS* routine. The non-hydrogen atoms were refined anisotropically and the hydrogen atoms were added to their geometrically ideal positions. The final formulas of **1** and **4** were determined by the single-crystal structures, element analysis results and TGA. The selected bonds lengths and angles are listed in Table S1.

**Table S1** Selected bond lengths [Å] and bond angles [º] for complexes **1**−**4**

| **Complex 1** | |  |  | | | |  | |
| --- | --- | --- | --- | --- | --- | --- | --- | --- |
| Cd(1)-N(2) | | 2.215(2) | N(2)#1-Cd(1)-N(1)#1 | | | | 71.27(8) | |
| Cd(1)-N(2)#1 | | 2.215(2) | N(2)-Cd(1)-N(1) | | | | 71.27(8) | |
| Cd(1)-N(1)#1 | | 2.407(3) | N(2)#1-Cd(1)-N(1) | | | | 108.73(8) | |
| Cd(1)-N(1) | | 2.407(3) | N(1)#1-Cd(1)-N(1) | | | | 180.00(12) | |
| Cd(1)-N(5)#2 | | 2.408(2) | N(2)-Cd(1)-N(5)#2 | | | | 91.13(7) | |
| Cd(1)-N(5)#3 | | 2.408(2) | N(2)#1-Cd(1)-N(5)#2 | | | | 88.87(7) | |
| N(2)-Cd(1)-N(2)#1 | | 180.0 | N(1)#1-Cd(1)-N(5)#2 | | | | 84.39(8) | |
| N(2)-Cd(1)-N(1)#1 | | 108.73(8) | N(1)-Cd(1)-N(5)#2 | | | | 95.61(8) | |
| N(2)-Cd(1)-N(5)#3 | | 88.87(7) | N(2)#1-Cd(1)-N(5)#3 | | | | 91.13(7) | |
| N(1)#1-Cd(1)-N(5)#3 | | 95.61(8) | N(1)-Cd(1)-N(5)#3 | | | | 84.39(8) | |
| N(5)#2-Cd(1)-N(5)#3 | | 180.0 |  | | | |  | |
|  |  | | |  | | |  | |
| **Complex 2** |  | | |  | | |  | |
| Cd(1)-O(6) | 2.272(4) | | | Cd(1)-N(1) | | | 2.267(5) | |
| Cd(1)-O(1) | 2.315(4) | | | Cd(1)-N(5) | | | 2.368(4) | |
| Cd(1)-O(2) | 2.434(5) | | | Cd(1)-N(4)#1 | | | 2.371(4) | |
| Cd(2)-O(5) | 2.287(4) | | | Cd(2)-N(6) | | | 2.276(4) | |
| Cd(2)-O(4) | 2.369(4) | | | Cd(2)-N(9)#2 | | | 2.348(4) | |
| Cd(2)-O(3) | 2.371(4) | | | Cd(2)-N(10) | | | 2.360(4) | |
| N(1)-Cd(1)-O(6) | 94.58(15) | | | N(1)-Cd(1)-O(1) | | | 108.85(14) | |
| O(6)-Cd(1)-O(1) | 156.45(15) | | | N(1)-Cd(1)-N(5) | | | 72.23(15) | |
| O(6)-Cd(1)-N(5) | 94.74(15) | | | O(1)-Cd(1)-N(5) | | | 94.71(15) | |
| N(1)-Cd(1)-N(4)#1 | 103.57(15) | | | O(6)-Cd(1)-N(4)#1 | | | 86.76(15) | |
| O(1)-Cd(1)-N(4)#1 | 85.43(15) | | | N(5)-Cd(1)-N(4)#1 | | | 175.63(18) | |
| N(1)-Cd(1)-O(2) | 158.62(14) | | | O(6)-Cd(1)-O(2) | | | 102.43(15) | |
| O(1)-Cd(1)-O(2) | 55.49(14) | | | N(5)-Cd(1)-O(2) | | | 93.32(16) | |
| N(4)#1-Cd(1)-O(2) | 90.36(16) | | | N(6)-Cd(2)-O(5) | | | 93.64(14) | |
| N(6)-Cd(2)-N(9)#2 | 102.26(15) | | | O(5)-Cd(2)-N(9)#2 | | | 88.93(15) | |
| N(6)-Cd(2)-N(10) | 71.89(15) | | | O(5)-Cd(2)-N(10) | | | 97.53(15) | |
| N(9)#2-Cd(2)-N(10) | 171.47(18) | | | N(6)-Cd(2)-O(4) | | | 161.85(14) | |
| O(5)-Cd(2)-O(4) | 98.89(14) | | | N(9)#2-Cd(2)-O(4) | | | 91.11(15) | |
| N(10)-Cd(2)-O(4) | 93.37(15) | | | N(6)-Cd(2)-O(3) | | | 113.73(14) | |
| O(5)-Cd(2)-O(3) | 152.54(14) | | | N(9)#2-Cd(2)-O(3) | | | 83.39(15) | |
| N(10)-Cd(2)-O(3) | 93.22(15) | | | O(4)-Cd(2)-O(3) | | | 55.15(13) | |
| N(6)-Cd(2)-H(5WA) | 78.6(12) | | | O(5)-Cd(2)-H(5WA) | | | 15.0(12) | |
| N(9)#2-Cd(2)-H(5WA) | 92.4(18) | | | N(10)-Cd(2)-H(5WA) | | | 92.5(18) | |
| O(4)-Cd(2)-H(5WA) | 113.4(12) | | | O(3)-Cd(2)-H(5WA) | | | 167.5(13) | |
|  |  | | | |  | | |  |
| **Complex 3** |  | | | |  | | |  |
| Cd(1)-O(1) | 2.329(2) | | | | Cd(1)-N(5)#1 | | | 2.363(2) |
| Cd(1)-N(4) | 2.367(3) | | | | Cd(1)-N(1) | | | 2.374(2) |
| Cd(1)-O(4) | 2.427(2) | | | | Cd(1)-O(3) | | | 2.445(2) |
| Cd(1)-O(2) | 2.5175(19) | | | |  | | |  |
| O(1)-Cd(1)-N(5)#1 | 91.06(8) | | | | N(5)#1-Cd(1)-O(3) | | | 101.89(8) |
| O(1)-Cd(1)-N(4) | 90.20(9) | | | | N(4)-Cd(1)-O(3) | | | 86.65(9) |
| N(5)#1-Cd(1)-N(4) | 171.35(8) | | | | N(1)-Cd(1)-O(3) | | | 122.04(8) |
| O(1)-Cd(1)-N(1) | 139.14(7) | | | | O(4)-Cd(1)-O(3) | | | 53.56(7) |
| N(5)#1-Cd(1)-N(1) | 103.93(8) | | | | O(1)-Cd(1)-O(2) | | | 53.66(7) |
| N(4)-Cd(1)-N(1) | 69.87(8) | | | | N(5)#1-Cd(1)-O(2) | | | 88.31(8) |
| O(1)-Cd(1)-O(4) | 140.48(7) | | | | N(4)-Cd(1)-O(2) | | | 85.54(8) |
| N(5)#1-Cd(1)-O(4) | 82.35(8) | | | | N(1)-Cd(1)-O(2) | | | 88.49(7) |
| N(4)-Cd(1)-O(4) | 102.00(8) | | | | O(4)-Cd(1)-O(2) | | | 162.80(7) |
| N(1)-Cd(1)-O(4) | 79.84(7) | | | | O(1)-Cd(1)-O(3) | | | 90.50(8) |
| O(3)-Cd(1)-O(2) | 143.20(7) | | | |  | | |  |
|  |  | | | | |  | |  |
| **Complex 4** |  | | | | |  | |  |
| Cd(1)-O(2) | 2.185(5) | | | | | Cd(2)-O(1) | | 2.321(4) |
| Cd(1)-O(4) | 2.232(5) | | | | | Cd(2)-O(3) | | 2.363(5) |
| Cd(1)-O(3) | 2.613(5) | | | | | Cd(2)-N(3)#2 | | 2.344(5) |
| Cd(1)-N(2) | 2.229(5) | | | | | Cd(2)-N(3) | | 2.344(5) |
| Cd(1)-N(5)#1 | 2.304(5) | | | | | Cd(2)-O(3)#2 | | 2.363(5) |
| Cd(1)-N(1) | 2.395(6) | | | | | Cd(2)-O(1)#2 | | 2.322(4) |
| O(2)-Cd(1)-N(2) | 101.37(19) | | | | | O(2)-Cd(1)-O(4) | | 134.8(2) |
| N(2)-Cd(1)-O(4) | 97.4(2) | | | | | O(2)-Cd(1)-N(5)#1 | | 89.16(19) |
| N(2)-Cd(1)-N(5)#1 | 159.8(2) | | | | | O(4)-Cd(1)-N(5)#1 | | 86.9(2) |
| O(2)-Cd(1)-N(1) | 117.4(2) | | | | | N(2)-Cd(1)-N(1) | | 71.75(19) |
| O(4)-Cd(1)-N(1) | 107.5(2) | | | | | N(5)#1-Cd(1)-N(1) | | 88.1(2) |
| O(2)-Cd(1)-O(3) | 90.22(18) | | | | | N(2)-Cd(1)-O(3) | | 80.89(17) |
| O(4)-Cd(1)-O(3) | 52.75(19) | | | | | N(5)#1-Cd(1)-O(3) | | 116.62(19) |
| N(1)-Cd(1)-O(3) | 144.01(19) | | | | | O(1)#2-Cd(2)-O(1) | | 180.0 |
| O(1)#2-Cd(2)-N(3)#2 | 87.67(18) | | | | | O(1)-Cd(2)-N(3)#2 | | 92.32(18) |
| O(1)#2-Cd(2)-N(3) | 92.33(18) | | | | | O(1)-Cd(2)-N(3) | | 87.67(18) |
| N(3)#2-Cd(2)-N(3) | 180.0 | | | | | O(1)#2-Cd(2)-O(3) | | 87.05(16) |
| O(1)-Cd(2)-O(3) | 92.96(16) | | | | | N(3)#2-Cd(2)-O(3) | | 90.96(19) |
| N(3)-Cd(2)-O(3) | 89.04(19) | | | | | O(1)#2-Cd(2)-O(3)#2 | | 92.95(16) |
| O(1)-Cd(2)-O(3)#2 | 87.05(16) | | | | | N(3)#2-Cd(2)-O(3)#2 | | 89.04(18) |
| N(3)-Cd(2)-O(3)#2 | 90.96(19) | | | | | O(3)-Cd(2)-O(3)#2 | | 180.0 |

Symmetry transformations used to generate equivalent atoms: for **1**, #1 -x+1, -y, -z+2, #2 -y+1/3, x-y-1/3, z+2/3, #3 y+2/3, -x+y+1/3, -z+4/3, #4 -x+y+2/3, -x+1/3, z-2/3; for **2**, #1 -x+1, -y, -z+1, #2 -x+1, -y, -z+2, #3 -x+1, -y+1, -z+1; for **3**, #1 -x+1, y-1/2, -z+5/2, #2 -x, y-1/2, -z+3/2, #3 -x+1, y+1/2, -z+5/2, #4 -x, y+1/2, -z+3/2; for **4**, #1 x, -y+1, z+1/2, #2 -x+1/2, -y+3/2, -z, #3 x, -y+1, z-1/2, #4 -x, -y+2, -z , #5 -x+1, y, -z+1/2.


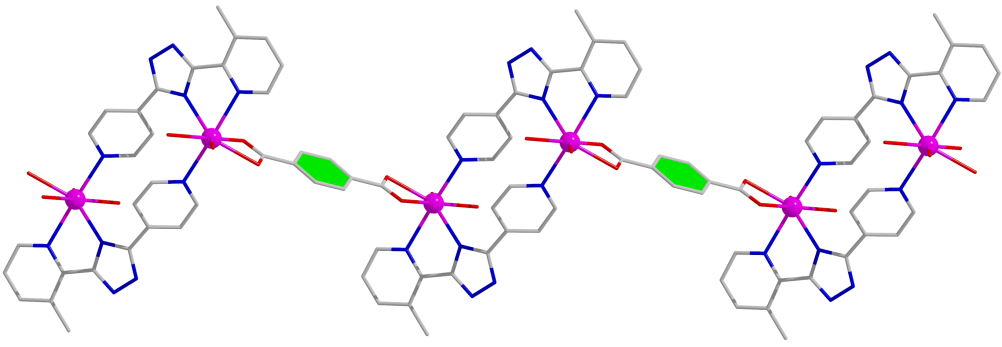


**Figure S1.** View of 1D chain of complex **3**.

**
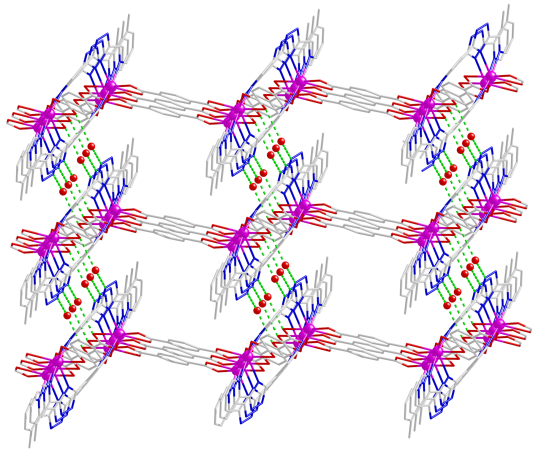
**

**Figure S2.** Inter-molecular hydrogen-bonding between the 2D layers yield a 3D suparmolecular with interlayer channels along *a* axis.


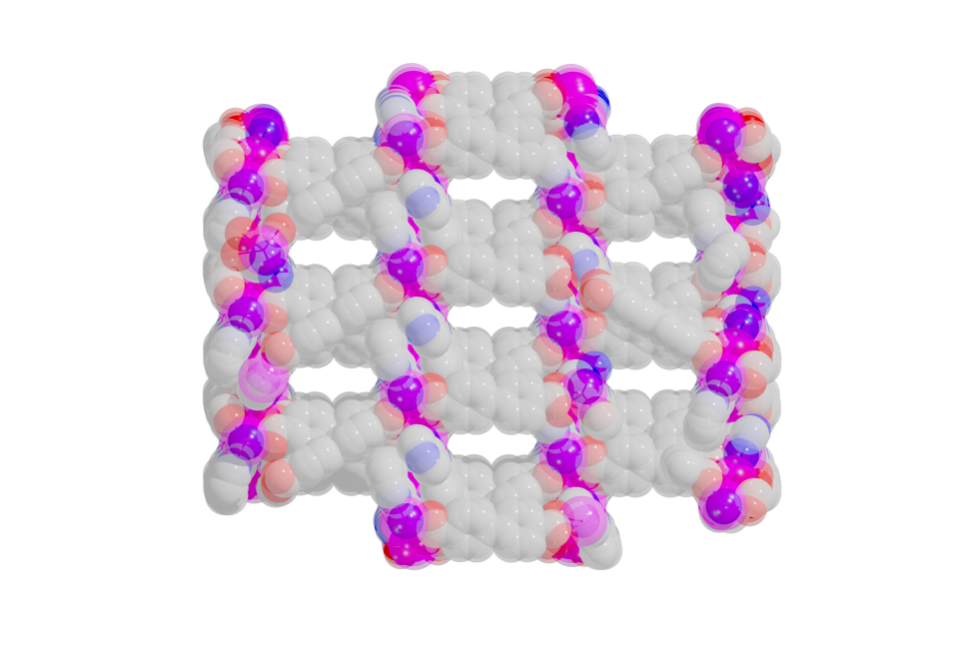


**Figure S3.** The 3D space-filling structure of **4** along *c* axis.


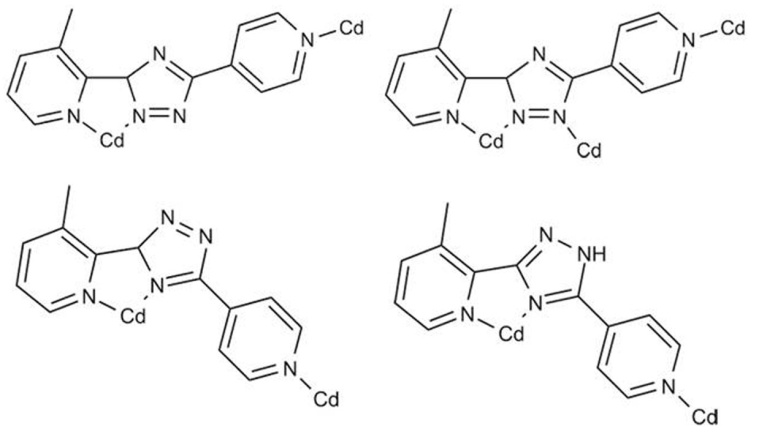


**Figure S4.** The coordination modes of Hmdpt/mdpt^-^ ligands.


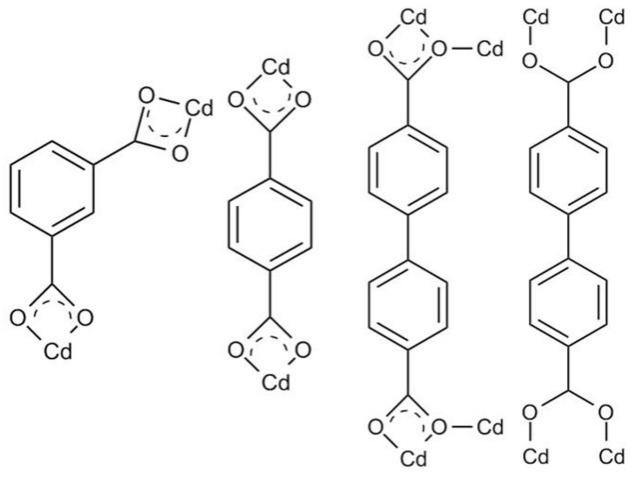


**Figure S5.** The coordination modes of different benzendicarboxylate ligands.


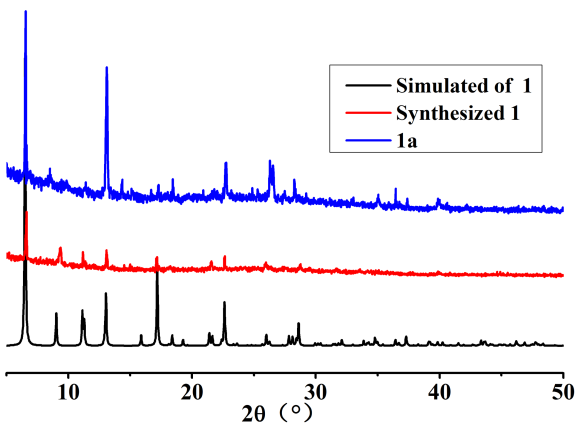

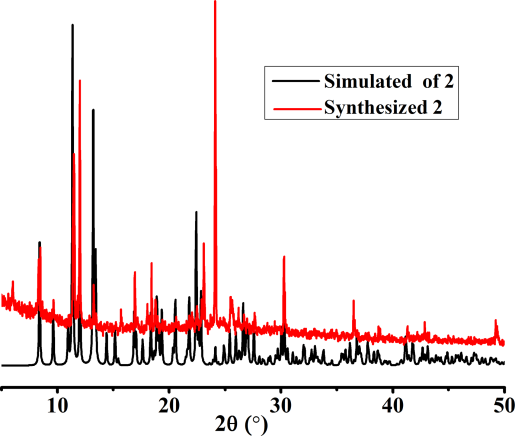


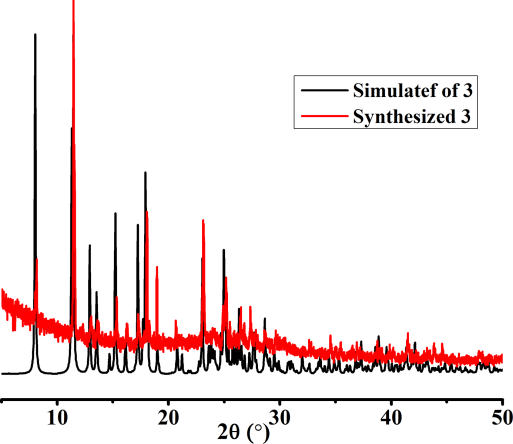

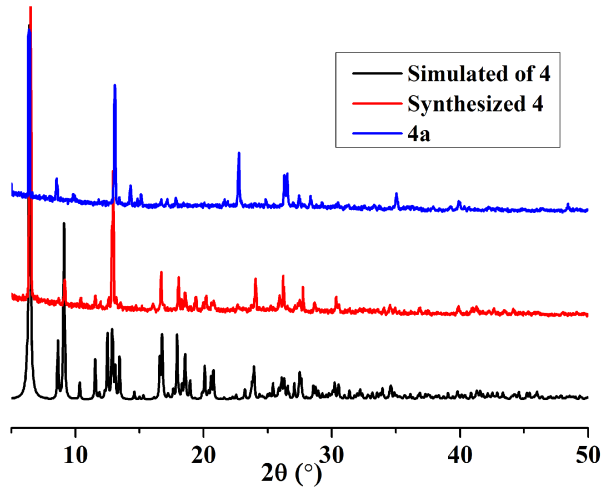


**Figure S6.** PXRD patterns of the as-synthesized products **1**−**4**.


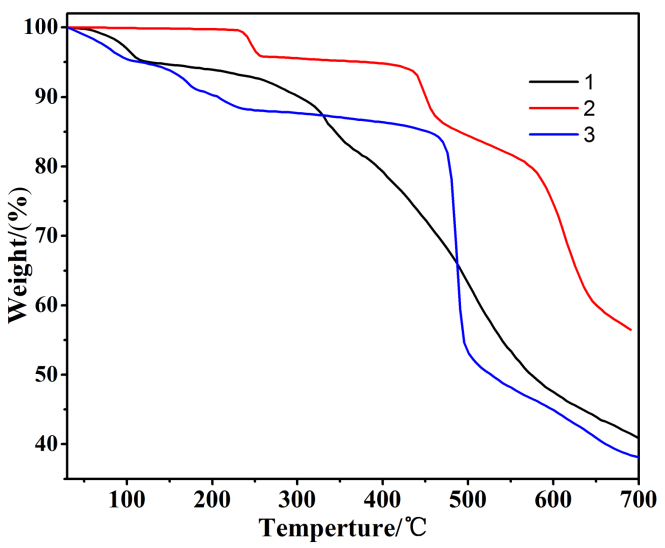

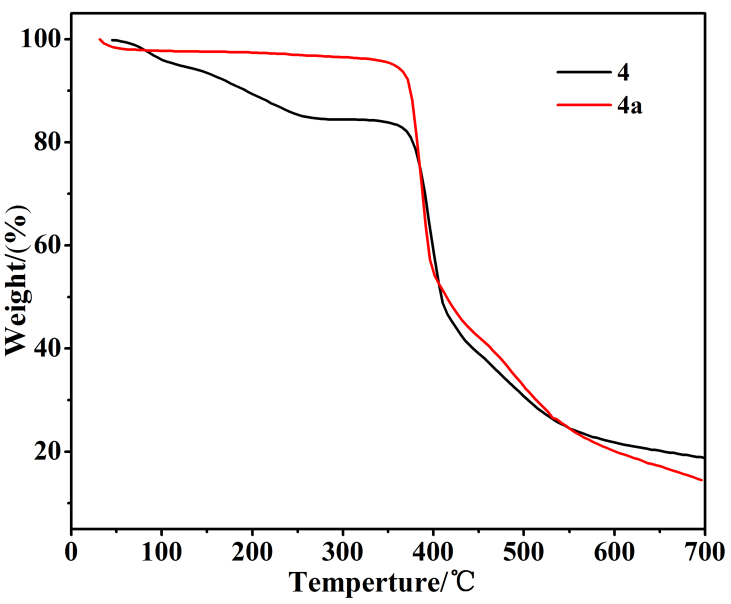


**Figure S7.** TGA curves for **1-4** and **4a** at the temperature 30-700 °C under N_2_ atmosphere.

**
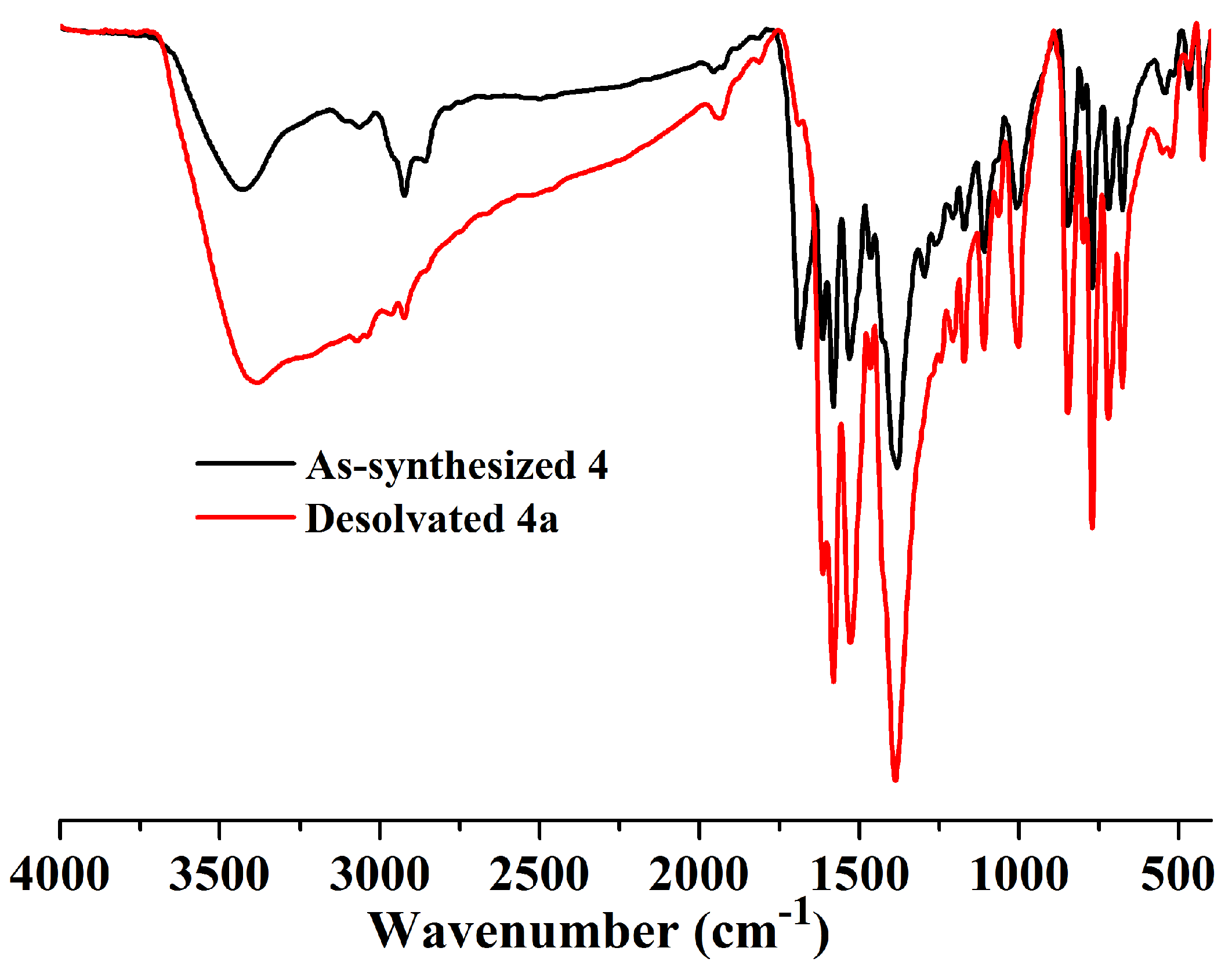
**

**Figure S8.** FT-IR spectra of the as-synthesized and desolvated **4** (**4a**). The characteristic C=O vibration at 1691 cm^-1^ of NMP in **4** is absent in **4a**, indicating the complete removal of NMP.

**
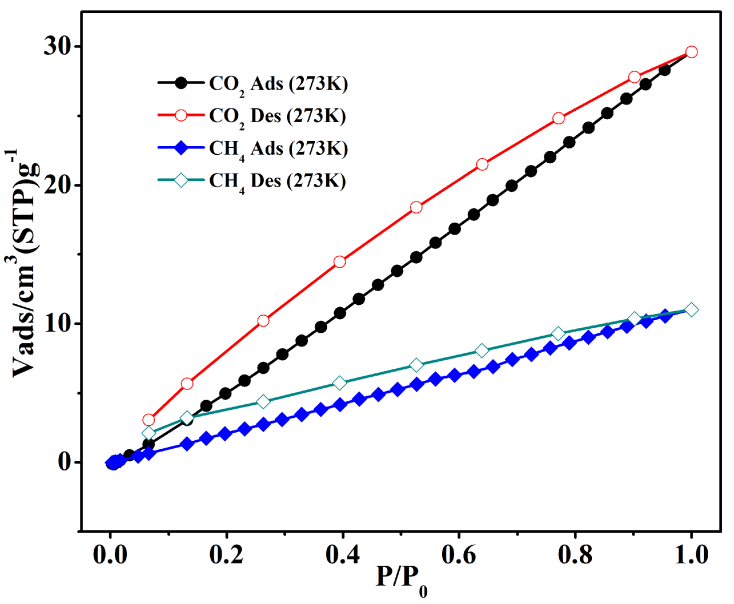
**

**Figure S9.** Gas sorption isotherms of **1** for CO_2_ and CH_4_ at 273 K.


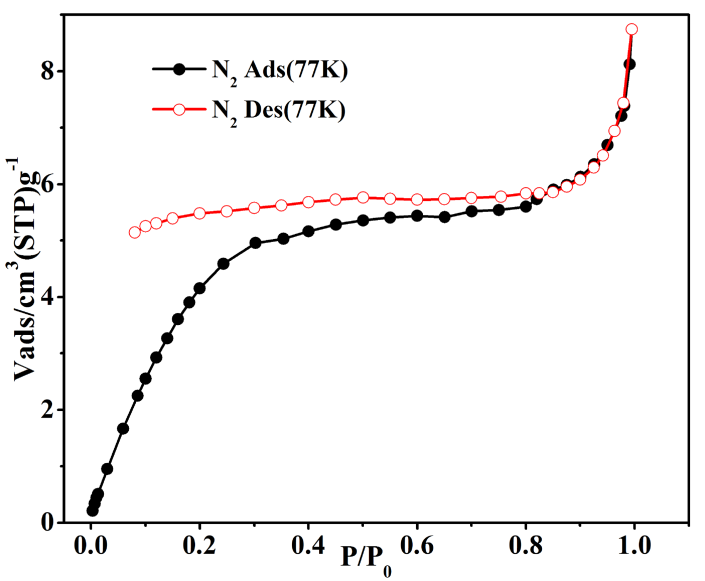


**Figure S10.** Gas sorption isotherms of **4a** for N_2_ at 77 k.

**IAST adsorption selectivity calculation**

The experimental isotherm data for pure CO_2_ and CH_4_ (measured at 273 and 298 K) were fitted using a Langmuir-Freundlich (L-F) model

$$q=\frac{a*b*p^{c}}{1+b*p^{c}}$$

Where *q* and *p* are adsorbed amounts and pressures of component *i*, respectively. The adsorption selectivities for binary mixtures of CO_2_/CH_4_ at 273 and 298 K, defined by

$$S_{ads}=\left(q1/q2\right)/\left(p1/p2\right)$$

Where *qi* is the amount of *i* adsorbed and *pi* is the partial pressure of *i* in the mixture.


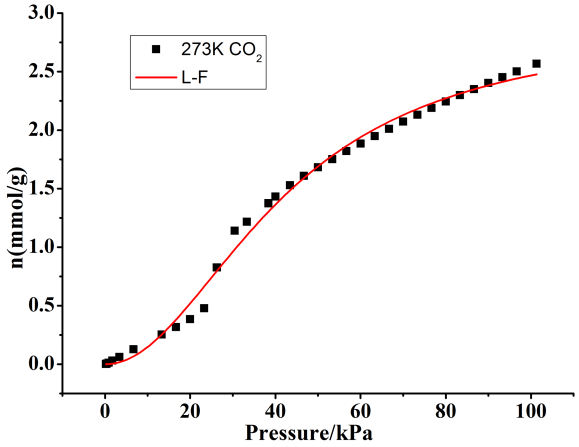

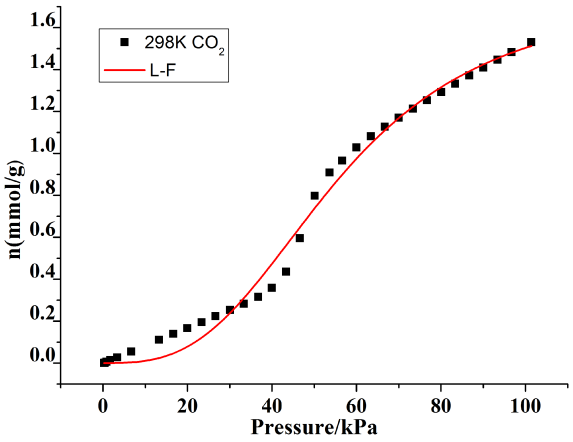


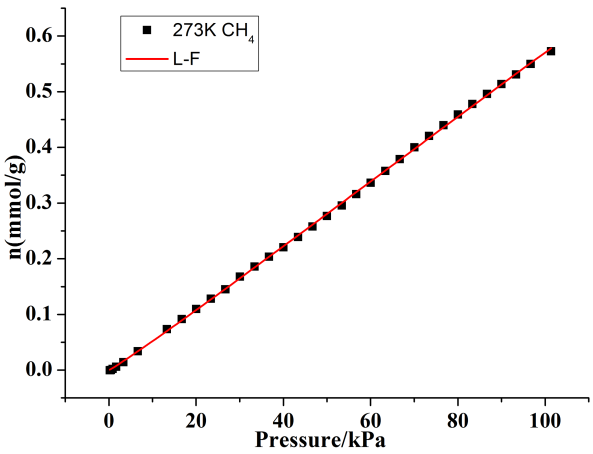

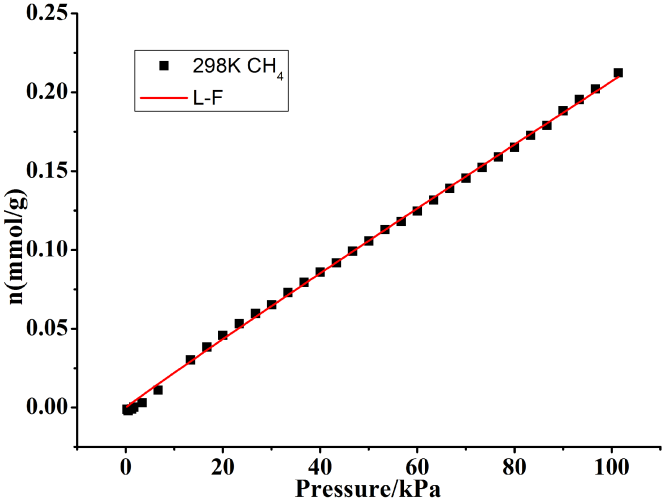


**Figure S11.** CO_2_ adsorption isotherms of **4a** at 273 K with fitting by L-F model: a =3.0455, b = 8.14512E-4, c = 1.86858, Chi^2 = 0.00175, R^2 = 0.99784; CO_2_ adsorption isotherms of **4a** at 298 K with fitting by L-F model: a = 1.77128, b = 6.10236E-6, c = 2.98171, Chi^2 = 0.00318, R^2 = 0.98967; CH_4_ adsorption isotherms of **4a** at 273 K with fitting by L-F model: a = 11.57008, b = 3.90455E-4, c = 1.06166, Chi^2 = 8.18069E-6, R^2 = 0.99976; CH_4_ adsorption isotherms of **4a** at 298 K with fitting by L-F model: a = 16.79683, b = 1.38495E-4, c = 0.97738, Chi^2 =4.78208E-6, R^2 = 0.99893.

**Calculation of sorption heat for CO_2_ and CH_4_ uptake using Virial 2 model**

The CO_2_ adsorption isotherm data for **4a** at 293 K was fitted using the Virial 2 expression, where *P* is the pressure, *N* is the adsorbed amount, *T* is the temperature, *a_i_* and *b_i_* are virial coefficients, and *m* and *N* are the number of coefficients used to describe the isotherms. *Q_st_* is the coverage–dependent enthalpy of adsorption and *R* is the universal gas constant.

$\ln p=\ln N+1/T\sum_{i=0}^{m} aiN^{i}+\sum_{i=0}^{n} biN^{i}$ $Q_{st}=-R\sum_{i=0}^{m} aiN^{i}$

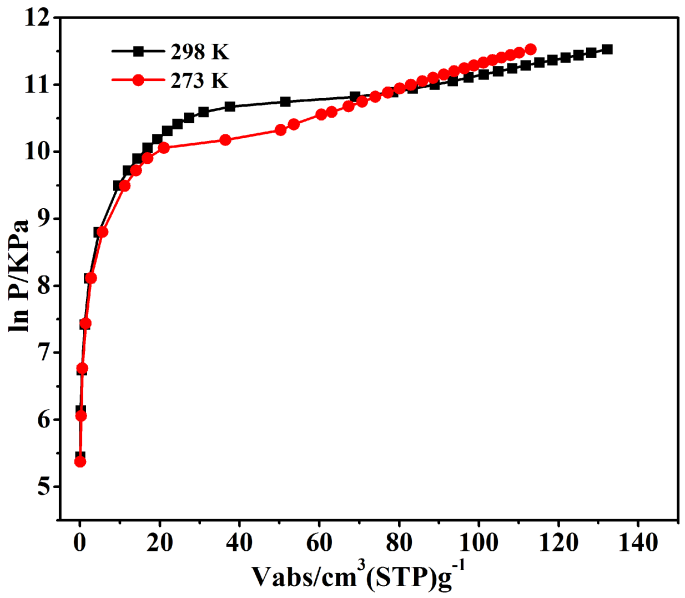


**Figure S12** CO_2_ adsorption isotherms for 1a with fitting by Virial 2 model. Fitting results: a0 = –2798.0597, a1 = 40.15582, a2 = -0.73603, a3 = 0.00291, b0 = 17.55597, b1 = -0.1614, b2 = 0.00227, Chi^2 = 0.01169, R^2 = 0.99548.


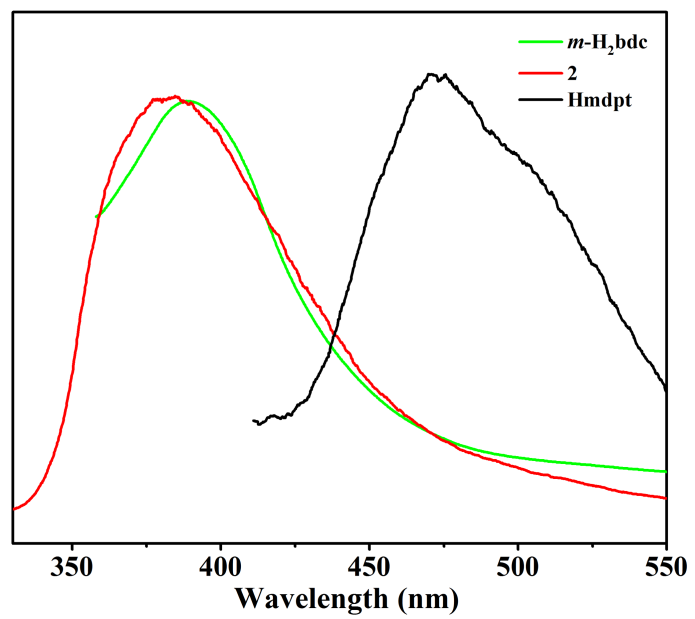

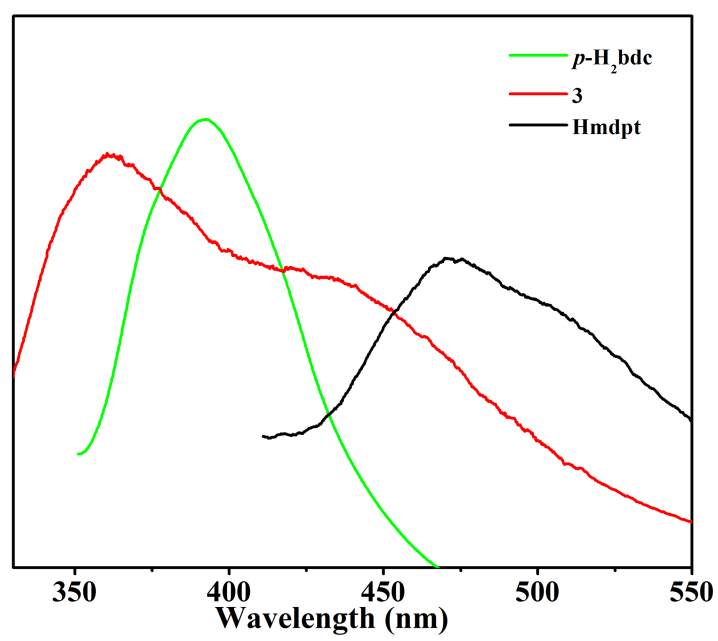


**
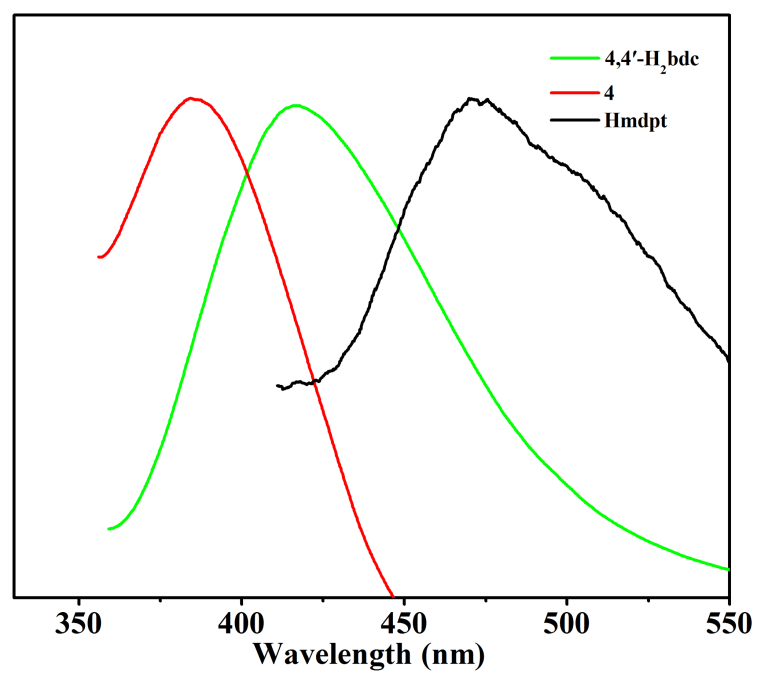
**

**Figure S13.** The luminescence properties of **2**–**4**, n-H_2_bdc and N-donor ligands.

**Table S2** the wavelengths of the emission (nm) of complexes **1**–**4**, Hmdpt and acid ligands.

|  | **Ligands** | | | |
| --- | --- | --- | --- | --- |
|  | Hmdpt | *m*-H_2_bdc | *p*-H_2_bdc | 4,4’-H_2_bpdc |
| λ_em_ | 472 | 388 | 392 | 416 |
|  | **complexes** | | | |
|  | **1** | **2** | **3** | **4** |
| λ_em_ | 425 | 380 | 360 | 386 |
